# Calmodulin Binding Domains in Critical Risk Proteins Involved in Neurodegeneration

**DOI:** 10.3390/cimb44110394

**Published:** 2022-11-21

**Authors:** Danton H. O’Day

**Affiliations:** 1 Department of Biology, University of Toronto Mississauga, Mississauga, ON L5L 1C6, Canada; danton.oday@utoronto.ca; 2 Department of Cell and Systems Biology, University of Toronto, Toronto, ON M5S 3G5, Canada

**Keywords:** calmodulin binding proteins, risk factor proteins, biomarkers, neurodegeneration, calmodulin hypothesis

## Abstract

Neurodegeneration leads to multiple early changes in cognitive, emotional, and social behaviours and ultimately progresses to dementia. The dysregulation of calcium is one of the earliest potentially initiating events in the development of neurodegenerative diseases. A primary neuronal target of calcium is the small sensor and effector protein calmodulin that, in response to calcium levels, binds to and regulates hundreds of calmodulin binding proteins. The intimate and entangled relationship between calmodulin binding proteins and all phases of Alzheimer’s disease has been established, but the relationship to other neurodegenerative diseases is just beginning to be evaluated. Risk factors and hallmark proteins from Parkinson’s disease (PD; SNCA, Parkin, PINK1, LRRK2, PARK7), Huntington’s disease (HD; Htt, TGM1, TGM2), Lewy Body disease (LBD; TMEM175, GBA), and amyotrophic lateral sclerosis/frontotemporal disease (ALS/FTD; VCP, FUS, TDP-43, TBK1, C90rf72, SQSTM1, CHCHD10, SOD1) were scanned for the presence of calmodulin binding domains and, within them, appropriate binding motifs. Binding domains and motifs were identified in multiple risk proteins, some of which are involved in multiple neurodegenerative diseases. The potential calmodulin binding profiles for risk proteins involved in HD, PD, LBD, and ALS/FTD coupled with other studies on proven binding proteins supports the central and potentially critical role for calmodulin in neurodegenerative events.

## 1. Introduction

Neurodegeneration leads to multiple psychological disorders from cognitive and emotional issues to altered social behaviors and ultimately dementia [1]. Anxiety, bipolar disorder, and schizophrenia are commonly expressed in dementia patients. Alzheimer’s disease (AD), amyotrophic lateral sclerosis (ALS), frontotemporal lobar dementia (FTD), Huntington’s disease (HD), and Parkinson’s disease (PD) are the most common neurodegenerative diseases and the aforementioned psychological effects, among others, are routinely observed [2,3]. Protein aggregation is a key pathogenic attribute of these diseases [4,5]. There is extensive evidence that toxic proteins and their resulting aggregates are involved in events underlying neuroinflammation and the disruption of cellular calcium levels. In fact, calcium dysregulation (calcium hypothesis) that characterises AD affects the function of multiple critical, experimentally validated calmodulin binding proteins (calmodulin hypothesis) that are central to every stage of the disease (e.g., AβPP1, BACE1, ADAM10, PSEN-1, Aβ, NMDAR, PMCA, RyR2, ABCA1, BIN1, Ng, CaMKII, PP2B, Tau, cdk25, and others) [6,7,8,9,10]. 

Initially, calmodulin (CaM) binding proteins (CaMBPs) were shown to be linked to the production of amyloid plaques and neurofibrillary tangles, the two hallmarks involved in the progressive neurodegenerative events leading to mild cognitive impairment and ultimately dementia. Subsequently, CaM was shown to regulate risk factor proteins, receptors, and critical proteins involved in neuroinflammation, not only in AD, but also in ALS, FTD, HD, PD, dementia with Lewy bodies (LBD), various tauopathies, and others [10]. To investigate this relationship further, one area that remained to be examined was the possible role of CaM in regulating recently identified risk and biomarker proteins associated with these neurodegenerative diseases.

Prior to this study, several risk factor proteins for HD, PD, LBD, ALS, and FTD had been examined for their proven and potential ability to bind to CaM. Huntingtin (HTT), which is the primary risk factor for HD, is an experimentally validated calcium-dependent CaMBP, and CaM binding increases with the length of the polyQ repeat [11]. CaM, HTT, and transglutaminase (TGM2) colocalize in vivo and co-immunoprecipitate. HTT is a substrate for both TGM2 and TGM1, enzymes that bind to and are activated by CaM [12,13,14]. Two CaMBPs involved in PD have been experimentally validated: D2 dopamine receptor and α-synuclein. CaM inhibits the D2-dopamine receptor (D2DR) by binding to a motif in the amino end of the protein involved in G-protein binding [15]. D2DR has been linked to schizophrenia as well as anxiety, co-morbid depression, and social dysfunction in veterans with post-traumatic stress disorder [16]. CaM also binds to both normal and mutant α-synuclein (SNCA), the main risk factor for PD and LBD, in a calcium-dependent manner in vitro and in vivo [17]. Several other critical proteins linked to neurodegenerative diseases have also been experimentally validated as CaMBPs, including APOE (AD, FTD, PD, LBD), calcineurin (PP2B; AD, FTD, PD, HD, MS), CaMKII (AD, FTD, PD, HD), and many others [10,18]. The potential involvement of CaMKII dysfunction in a myriad of neuropsychiatric disorders including depression, epilepsy, and schizophrenia, as well as neurodevelopmental disorders has long been recognized [19].

To gain additional insight into the role of calmodulin in neurodegeneration, a number of recently identified risk factors were analyzed for their potential ability to bind to CaM. Two proteins cited as risk factors for LBD were evaluated: TMEM175 (Transmembrane protein 175; also PD) and GBA (glucocerebrosidase, a lysosomal enzyme; also PD) [20]. SNCA, Parkin, PINK1, LRRK2, and PARK7 that have been categorized as risk and/or marker proteins for PD were analyzed for their potential CaM-binding ability [21,22]. FUS and TPD-43 (TARDBP), marker and risk proteins linked to FTD that accumulate in the frontal and temporal lobes, were evaluated, as well as risk proteins that overlap between FTD and ALS including SQSTM1, VCP, TBK1, CHCHD10, C9orf72, and progranulin (GRN) [23,24]. Along with FUS and TDP-43, SOD1, which contributes to inclusions in ALS neurons, was also evaluated [23]. The risk proteins for HD that were analyzed were huntingtin and TGMs 1-3. 

## 2. Results

### 2.1. CaMBDs of Parkinson’s Marker Proteins

Despite the experimental verification of CaM binding to SNCA and its apparent significance, the location or nature of the CaMBD in this protein remains undetermined [17]. A Calmodulin Target Database scan revealed one extensive calcium dependent binding region (32KTKQGVAEAAGKTKEGVLYVGSKTKEGVVH50) within which two 1-12 binding motifs are present (Table 1). While Parkin (E3 ubiquitin-protein ligase parkin) mutations are the most common cause of familial and sporadic early onset PD, a scan of human Parkin did not reveal any putative CaMBD. In contrast, the two PINK1 isoforms each demonstrated CaMBDs with multiple binding motifs. Isoform 1 (72RQSVAGLAARLQRQFVVRAW90) possesses four binding motifs (two 1-5-10; 1-8-14; 1-14), while isoform 2 (211ALKNLKLDKMVGWLLQQSAA230) has three binding motifs (two 1-10; 1-14; Table 1). Leucine-rich repeat serine/threonine-protein kinase 2 (LRRK2) possesses a single canonical 20-aa CaMBD at position 776-795, with four binding motifs (1-10, 1-14, 1-12, 1-16; Table 1). Analysis of human Parkinson disease protein 7 (PARK7) revealed a single 18-aa CaMBD (93KEQENRKGLIAAICAGPT110) with a non-canonical binding motif, in which a cluster of six hydrophobic amino acids is present (Table 1).

### 2.2. Dementia with Lewy Bodies: CaM-Binding of TMEM75 and GBA

Three LBD risk factor/biomarker proteins (BIN1, SNCA, APOE) have previously been identified as proven or putative CaMBPs [9,10]. In this study, a scan revealed TMEM175 contains a single calcium-dependent CaMBD (172IQRSAHRALYRRHVLGIVL190) that Uniprot reveals is present in a cytoplasmic region of the protein (Table 1). This domain contains 5 different binding motifs (1-5-10, 1-14, two 1-10, 1-12). The lysosomal enzyme GBA also contains one identified calcium-dependent CaMBD (249ARYFVKFLDAYAEHKLQFW268) with three binding motifs (1-12, 1-16, 1-10), plus a calcium-independent IQ motif (204LQLAQRPVSLLASP217; Table 1).

### 2.3. CaM-Binding of ALS/FTD Risk Proteins

A Calmodulin Target Database scan revealed that four of the seven risk proteins for ALS/FTD contain a single calcium-dependent CaMBP, while one has an IQ-like domain. VCP (valosin-containing protein) shows a single eight amino acid potential CaMBP (738EAMRFARR745) but, as a stand-alone sequence, it appears too short and lacks sufficient hydrophobic amino acids to be a canonical CaMBD. While SQSTM1 (sequestosome-1) and CHCHD10 (coiled-coil-helix-coiled-coil-helix domain-containing protein 10) have no presumptive calcium-dependent CaMBDs, SQSTM1 possesses an IQ motif. On the other hand, the FUS (fused in sarcoma) 19 aa CaMBD (301VADYFKQIGIIKTNKKTGQ319) contains a single 1-5-10 motif, while the same length TDP-43 (TAR DNA-binding protein 43; TDP-43) CaMBP (135VKKDLKTGHSKGFGFVRFT153) that was detected possesses three binding motifs (1-16, 1-12, 1-14; Table 2). The CaMBD of TBK1 (TANK-binding kinase 1) contains five potential binding motifs (22NVFRGRHKKTGDLFAIKVF40; two 1-12, two 1-14, 1-16), while C9orf72 (chromosome 9 open reading frame 72) had a 20 aa CaMBD (241AEKVNKIVRTLCLFLTPAER260), displaying a 1-5-10 and a 1-12 binding motif (Table 2). In contrast, no CaMBDs were detected in either SOD1 or GRN.

### 2.4. CaM-Binding Domains in Huntington Risk Proteins

Both HTT and TGM2 have been experimentally verified as CaMBPs, but their potential binding domains remain to be identified [13,25,26]. A Calmodulin Target Database search was carried out for both. Human HTT (P42858) shows two CaMBDs: CaMBD1 (177NGAPRSLRAALWRFAELAHLVR197) containing 3 motifs (1-12, 1-8-14, 1-5-10) and CaMBD2 (2535PLKALDTRFGRKLSIIRGIV2554) with 5 motifs (three 1-12, 1-8-14, 1-16; Table 3). Two CaMBDs were detected in human TGM2 (P21980), while a single CaMBD was identified in TGM1 (Table 3). TGM2 CaMBD1 (414KSINRSLIVGLKISTKSVGR433) contained 3 motifs (1-16, 1-12, 1-5-10), while CaMBD2 (665VVNFESDKLKAVKGFRNVII683) displayed 5 motifs (two 1-12, 1-8-14, two 1-10). The TGM1 CaMBD (607RRTVKLHLYLSVTFYTGVS625) showed 2 motifs (1-12, 1-5-10). TGM3 had a single CaMBD (493LAVGKEVNLVLLLKNLSRDT512) with 5 binding motifs (1-10, 1-12, 1-16, two 1-5-10, 1-14; Table 3). These results are in line with the CaM binding ability of the three proteins, but the sequences and motifs require experimental validation.

## 3. Discussion

Protein domains underlie the structure and function of proteins, therefore identifying them is a critical step in protein analysis [27]. As fundamental units, their detection is central to understanding not only protein function, but also protein evolution. There are many online predictive resources for determining highly conserved domains in proteins [27]. In contrast, defining the presence of calmodulin binding domains presents unique challenges [28,29]. Unlike typical domains that consist of a fairly rigorous arrangement of amino acids, there are many and variable types of calmodulin binding domains [30,31,32]. Calcium-independent calmodulin binding domains are characterized by IQ or IQ-like domains, in which proteins bind to apo-calmodulin via amino acid sequences beginning with a hydrophobic amino acid next to a glutamine residue with optional downstream acidic residues in appropriate positions. Classical binding to calcium-charged calmodulin occurs via a diversity of sequences involving multiple hydrophobic residues with various spacings and often the intervention of acidic residues. In addition to the dozen or so canonical binding domains, many novel and bipartite sequences have also been revealed [33]. Using profile hidden Markov model algorithms to predict CaM binding proteins (CaMBPs), the Calmodulin Target Database stands out as the “gold standard” for the prediction of canonical CaM-binding domains in proteins longer than 100 amino acids [28,29,34]. The Calmodulin Target Database has a greater than 80% prediction rate for identifying valid canonical CaMBDs and this accuracy is increased when putative CaMBPs show multiple overlapping canonical motifs [29]. In this study, identified CaMBDs were then visually scanned to assess and categorize binding motifs detected within them [10,18]. IQ and IQ-like motifs that mediate calcium-independent CaM binding were also found by visual scanning of protein sequences.

As a major cause of the diversity of psychological disorders during the early disease stages and subsequent dementia, understanding the underlying events of neurodegeneration is critical. Calmodulin is a common denominator in all aspects of Alzheimer’s disease, the primary neurodegenerative cause of dementia, from the generation of the classic hallmarks—amyloid beta plaques and neurofibrillary tangles—to the regulation of critical receptors and biomarkers [8,9,10]. The calmodulin hypothesis, an extension of the calcium hypothesis of Khachaturian, has been shown to be applicable to neuroinflammation in AD, HD, PD, LBD, and other neurodegenerative diseases [6,7,10]. Neuroinflammation has been linked to several psychological disorders including anxiety, depression, and schizophrenia [35]. The analyses here have shown that many key marker and risk proteins for HD (Huntingtin, TGM1, TGM2), PD (α-synuclein, PINK1, LRR2, PARK7), LBD (TMEM175, GBA), and ALS/FTD (TDP-43, SQSTM1, FUS, TBK1, C9orf72) all possess identifiable CaMBPs each with one or more binding motifs. 

After AD, the second most common neurodegenerative disease is PD. It is characterized by the loss of nigrostriatal dopaminergic (DA) neurons, decreased dopamine levels, and the presence of SNCA-rich Lewy bodies (LB) that are found in both sporadic and familial PD. PD differs from other synucleinopathies (e.g., LBD; multiple system atrophy (MSA)) in the primary localization of LBs in DA neurons present in the substantia nigra pars compacta region of the brain. Aβ, tau, and α-synuclein are all found in Lewy bodies in both PD and LBD [36,37]. A recent review of CaMBPs in PD focused on experimentally validated CaMBPs, including CaMKII, calcineurin, NMDAR, AchR, Adenosine A2AR, and cdk5, but failed to mention or assess the CaM-binding ability of other critical PD proteins, including the risks and biomarkers covered here [38]. The involvement of CaMKII and A2AR in psychiatric disorders was discussed above. In addition, dysfunctional NMDARs have been implicated in various neuropsychiatric disorders, including autism spectrum disorder, epilepsy, and schizophrenia [39]. AchR dysfunction is also involved in schizophrenia [40]. In addition to the proteins covered in the review, several other PD-linked CaMBPs should be noted. Aβ and tau, which both contribute to LBs, are experimentally proven CaMBPs [36,41] [42]. A beta-secretase (BACE1) polymorphism is also associated with PD [43]. BACE1 was originally identified as a potential CaMBP using the methods described in this article [8]. Using that information, CaM was subsequently shown not only to bind to BACE1, but also to increase its enzyme activity 2.5-fold, an event that was inhibited by five different CaM antagonists [44]. In contrast, CaM inhibits the D2-dopamine receptor, a critical factor in PD, by binding to a motif in the amino end of the protein that is involved in G-protein binding [15]. The well-characterized CaMBP neurogranin (Ng, RCE, p17, BICKS) is not only a useful biomarker for AD, but also for PD, as well as Creutzfeldt–Jakob disease and other brain diseases [45]. Several other CaMBPs include CaMKII and calcineurin plus two PD-associated proteins, DnaJ heat shock protein family member C13 (DNAJC13) and the GTP-binding protein RIT2 [37]. 

CaM binds to both normal and mutant SNCA in a calcium-dependent manner in vitro and in vivo and plays functions in LB formation by enhancing the SNCA fibril formation [17,46]. Aggregation of SNCA leads to calcium dyshomeostasis disrupting downstream signal transduction including CaM-mediated events [47,48]. While SNCA was previously identified as a CaMBP, its binding domains were not determined. Here we have identified a likely calcium-dependent CaMBD (32KTKQGVAEAAGKTKEGVLYVGSKTKEGVVH50) within SNCA that contains two 1-12 binding motifs. In addition, three other risk or biomarker proteins (PARK7, LRRK2, PINK1) possess potential binding domains. PINK1 has two binding domains each with multiple binding motifs, while LRRK2 has a single identified CaMBD with multiple binding motifs. PARK7 appears to possess a non-canonical CaMBD.

Dementia with Lewy bodies (LBD) and PD share many biochemical, clinical, pathological, and genetic risk factors. Despite this, LBD is differentiated from AD and PD on memory deficiencies, motor impairments, and sleep issues, among others. Still, a number of risk factors for LBD are linked to other neurodegenerative diseases: BIN1 (also AD), TMEM175 (Transmembrane protein 175; also PD), SNCA (also PD), APOE (also AD), and GBA (glucocerebrosidase; also PD, Gaucher’s disease) [10,20]. GBA, a lysosomal enzyme involved in lipid degradation, cholesterol metabolism, and membrane turnover, functions in microglial differentiation and movement and serves as a negative regulator of neuroinflammation. Mutations in *GBA1*, the gene encoding GBA, are linked to an increased risk of PD, LBD, and other synucleinopathies [49,50,51]. The mutations decrease GBA activity, enhancing the accumulation of SNCA [50]. Decreased GBA activates microglia, leading to neuroinflammation [49]. GBA also binds to SNCA with this interaction occurring via C-term residues 118-137, which differs from the CaMBD assessed above [46]. The Calmodulin Target Database failed to reveal a CaMBD in this region, but a visual scanning of this basic amino acid-rich region revealed a potential CaMBD (118KGFGGAMTDAAALNILALSP127) with four binding motifs. This suggests GBA may have three CaMBDs of which only two were detected by database analysis.

The comparatively less well-studied frontotemporal dementia (FTD) with and without Pick’s bodies (aka Pick’s disease) is also characterized by CaMBPs. FTD is a rare form of dementia that affects individuals younger than 65 [23]. With a diverse symptomology and occurring in less than 5% of dementia cases, FTD can be inherited, but spontaneous forms of the disease occur more often. Many proteins linked to FTD that accumulate in the frontal and temporal lobes include tau, progranulin (GRN), FUS, and TPD-43. Several risk and marker proteins overlap between FTD and ALS: TDP-43, SQSTM1, VCP, FUS, TBK1, CHCHD10, and C9orf72 [23]. While ALS and FTD exhibit specific attributes, they are discussed together here for these reasons and due to limited space. TDP-43, FUS, TBK1, and C9orf72 were all found to contain at least one calcium-dependent CaMBD with one or more binding motifs. SQSTM1 alone possesses a calcium-independent IQ-like binding motif, while VCP lacked any identifiable CaMBD. SQSTM1 has also been linked to other neurodegenerative diseases including AD, PD, and HD [52]. Pick’s bodies are filamentous aggregations of the phosphorylated protein tau, and the amount of tau correlates with the degree of disease symptoms. Different patterns of tau phosphorylation from that seen in AD lead to different filamentous organization in Pick’s bodies [53]. Calmodulin-like skin protein (CLSP; aka calmodulin-like 5), which has four EF-hand domains and is functionally similar to CaM, is a potential biomarker for Pick’s disease [54]. Originally linked to epidermal keratinocyte differentiation, CLSP also colocalizes with tau in Pick’s bodies and binds to the CaMBP transglutaminase 1 (TGM1) [14]. 

HD is a monogenic neurological disease caused by a CAG (cytosine-adenine-guanine) repeat mutation that generates a glutamine (Q) homopolymer stretch of residues in the Huntington (HTT) protein. While other factors are in play, the length of the polyQ homopolymer is the major determinant for the timing of disease onset. HTT is a calcium-dependent CaMBP and CaM binding increases with the length of the polyQ repeat [11]. CaM, HTT, and transglutaminases (TGM1-3) colocalize in vivo and co-immunoprecipitate. The relationship between HTT and TGM2 has been studied the most. HTT is a substrate for TGM2, an enzyme that binds to and is activated by CaM [12]. Furthermore, CaM regulates the cross-linking of HTT that is mediated by TGM2 [13]. Other neurodegenerative diseases, including AD and PD, also show increased levels of TGM2 [55]. Despite the aforementioned studies, the CaMBDs of HTT and TGM2 were not identified in those studies and Uniprot does not list CaM binding as an attribute for either of these two proteins. In this study. we’ve shown that HTT has two potential CaMBDs (CaMBD1, 177NGAPRSLRAALWRFAELAHLVR197; CaMBD2, 2535PLKALDTRFGRKLSIIRGIV2554), each with multiple binding motifs. TGM2 also has two binding domains CaMBD1, 414KSINRSLIVGLKISTKSVGR433; CaMBD2, (665VVNFESDKLKAVKGFRNVII683), each with multiple binding motifs, while TGM1 has a single CaMBD (607RRTVKLHLYLSVTFYTGVS625) with two motifs. TGM3 also has a single CaMBD (493LAVGKEVNLVLLLKNLSRDT512), but with five binding motifs.

**Figure 1:**
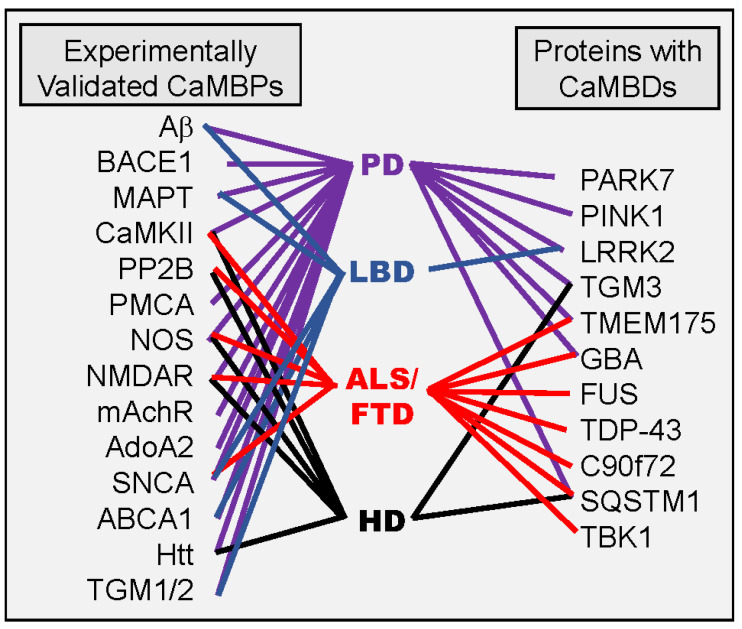
Experimentally validated (left side) and putative (right side) calmodulin binding proteins that are risk factors or biomarkers for specific neurodegenerative diseases are revealed by the connecting lines. See text for details.

It has previously been shown that numerous experimentally validated CaMBPs are involved in multiple neuroinflammatory events including Aβ (AD, PD, MS), BACE1 (AD, PD), BIN1 (AD, LBD), CaMKII (AD, PD, HD. FTD), calcineurin (AD, PD, HD. ALS, MS), NMDAR (AD, PD, HD. FTD), AchR (AD, PD), NOS (AD, PD, HD. ALS), and more (Figure 1) [10]. Some of the experimentally validated and putative CaMBP risk proteins analyzed here for PD, HD, LBD, and ALS/FTD also are interlinked (Figure 1). For example, the LBD risk factors GBA and TMEM175 have also been linked to ALS/FTD and PD [56]. SQUSTM1, an ALS/FTD risk factor, has been shown to be involved in both HD and PD [52]. The HD risk proteins HTT and TGM2 are also involved in PD, while LRRK2 and SNCA have been linked to LBD [57,58]. While not discussed here, many of these risk factor CaMBPs are also associated with AD.

Research into calmodulin-based therapies to treat neurodegenerative diseases is already underway but has a long way to go. Polysialic acid-based micelles that effectively cross the blood–brain barrier were used to deliver the CaM antagonist DY-9836 to treat vascular dementia with resulting cognitive improvements [59]. Inhibitors of the CaM-binding proteins CaMKII and CaN have long been known to reduce plaque burden, restore memory deficits, and even reduce the incidence of dementia [60,61,62]. Numerous pharmaceuticals are available that not only target specific calmodulin binding proteins, but also specific states of those CaMBPs. AS105, GS-680, and RA306 are ATP-competitive inhibitors of activated CaMKII, while KN-93 is an allosteric inhibitor of CaM binding to inactive CaMKII [63]. Currently, drugs that affect more than one target (e.g., BACE1 and AchR) have been developed, suggesting that this approach could be viable for co-targeting more than one specific CaMBP [64]. Other approaches to regulating CaMKII function (e.g., antisense oligonucleotides, small interfering RNA, and miRNAs) are also under analysis [63]. The key here is that calmodulin and its binding proteins lie at the heart of multiple neurodegenerative diseases and that multiple drugs and novel approaches exist, suggesting that the time is ripe to begin serious therapeutic research in this area.

As with any domain or motif detected through consensus sequence modeling, experimental verification is required. The Calmodulin Target Database is acknowledged as the best method for predicting CaM-binding [29,34]. Its use here has clearly revealed CaMBDs in a diversity of risk proteins associated with HD, PD, LBD, ALS, and FTD. Based on its accuracy of CaMBP detection (~80%) and the significance of multiple binding domains mentioned above, this would suggest the following proteins as priority candidates for further investigation: PD (PINK1, LRRK2), LBD (TMEM175, GBA), ALS/FTD (TDP43, TBK1), and HD (Huntingtin, TGM2, TGM3) [29,65]. Coupled with earlier studies showing CaM binding to critical proteins involved in neurodegenerative diseases, these results point to calmodulin as a critical therapeutic target in many, if not all, neurodegenerative diseases.

## 4. Materials and Methods

The proteins for this study were selected from recent research studies on risk factors and hallmark proteins from HD (Htt, TGM1, TGM2), PD (SNCA, Parkin, PINK1, LRRK2, PARK7), LBD (TMEM175, GBA), and ALS/FTD (VCP, FUS, TDP-43, TBK1, C90rf72, SQSTM1, CHCHD10, SOD1). Since ALS and FTD share these risk genes, we consider them together for this report [23]. After a search of the literature for evidence that any of these proteins demonstrated experimentally validated CaM-binding, the remaining protein sequences from the Uniprot database (www.uniprot.org) were scanned for CaMBDs using the Calmodulin Target Database [9,10,18,65]. The identified CaMBDs were then visually scanned to identify binding motifs within them as previously detailed [9,66]. There is no existing computer algorithm that can perform these time-consuming but important analyses. Datasets are not required since the raw data and analyses are presented in Table 1, Table 2 and Table 3. Multiple proteins linked to AD that were identified as CaMBPs using this approach have subsequently been experimentally validated as CaMBPs (BACE1, PSEN-1, AβPP1) [44,67,68].

## Figures and Tables

**Table 1:** Calmodulin binding domains and motifs in Parkinson’s and Lewy body dementia risk proteins.

Protein/Uniprot	CaMBD Sequence/Binding Motif
**Parkinson’s Disease**	
	** 1 12**
**SNCA/**	**32KTKQGVAEAAGKTKEGVLYVGSKTKEGVVH50**
**P37840**	** 1 12**
	**32KTKQGVAEAAGKTKEGVLYVGSKTKEGVVH50**
	** 1 8 14**
**PINK1/isoform 1**	**72RQSVAGLAARLQRQFVVRAW90**
**Q9BXM7**	** 1 5 10**
	**72RQSVAGLAARLQRQFVVRAW90**
	** 1 14**
	**72RQSVAGLAARLQRQFVVRAW90**
	** 1 5 10**
	**72RQSVAGLAARLQRQFVVRAW90**
	** 1 10**
**PINK1/isoform 2**	**211ALKNLKLDKMVGWLLQQSAA230**
**Q9BXM7**	** 1 14**
	**211ALKNLKLDKMVGWLLQQSAA230**
	** 1 10**
	**211ALKNLKLDKMVGWLLQQSAA230**
	** 1 10**
**LRRK2/**	**776TISIGKGDSQIISLLLRRLA795**
**Q5S007**	** 1 14**
	**776TISIGKGDSQIISLLLRRLA**
	** 1 12**
	**776TISIGKGDSQIISLLLRRLA**
	** 1 16**
	**776TISIGKGDSQIISLLLRRLA**
	**non-canonical:**
**PARK7/**	**93KEQENRKGLIAAICAGPT110**
**Q99497**	
**Lewy Body Dementia**	
	** 1 5 10**
**TMEM175/**	**172IQRSAHRALYRRHVLGIVL190**
**Q9BSA9**	** 1 14**
	**172IQRSAHRALYRRHVLGIVL190**
	** 1 10**
	**172IQRSAHRALYRRHVLGIVL190**
	** 1 12**
	**172IQRSAHRALYRRHVLGIVL190**
	** 1 10**
	**172IQRSAHRALYRRHVLGIVL190**
	** 1 12**
**GBA/**	**249ARYFVKFLDAYAEHKLQFW268**
**P04062**	** 1 16**
	**249ARYFVKFLDAYAEHKLQFW268**
	** 1 10**
	**249ARYFVKFLDAYAEHKLQFW268**
	** 1 12**
**^1^GBA/**	**32KTKQGVAEAAGKTKEGVLYVGSKTKEGVVH50**
**P04062**	** 1 14**
	**32KTKQGVAEAAGKTKEGVLYVGSKTKEGVVH50**
	** 1 10**
	**32KTKQGVAEAAGKTKEGVLYVGSKTKEGVVH50**
	** 1 12**
	**32KTKQGVAEAAGKTKEGVLYVGSKTKEGVVH50**
**^1^GBA IQ motif**	**204LQLAQRPVSLLASP217**

Legend: Green, critical hydrophobic amino acids; TMEM175, transmembrane protein 175; GBA, beta-glucocerebrosidase; LRRK2, Leucine-rich repeat serine/threonine-protein kinase 2; PARK7, Parkinson disease protein 7; PINK1, Serine/threonine-protein kinase PINK1; SNCA, a-Synuclein. ^1^CaMBD determined by visual assessment (See Discussion).

**Table 2:** Calmodulin binding domains and motifs in proteins linked to ALS/FTD.

Protein/Uniprot	CaMBD Sequence/Binding Motif
**FUS/**	** 1 5 10**
**P35637**	**301VADYFKQIGIIKTNKKTGQ319**
**TDP43/**	** 1 16**
**Q13148**	**135VKKDLKTGHSKGFGFVRFT153**
	** 1 12**
	**135VKKDLKTGHSKGFGFVRFT153**
	** 1 14**
	**135VKKDLKTGHSKGFGFVRFT153**
**TBK1/**	** 1 12**
**Q9UHD2**	**22NVFRGRHKKTGDLFAIKVF40**
	** 1 14**
	**22NVFRGRHKKTGDLFAIKVF40**
	** 1 12**
	**22NVFRGRHKKTGDLFAIKVF40**
	** 1 14**
	**22NVFRGRHKKTGDLFAIKVF40**
	** 1 16**
	**22NVFRGRHKKTGDLFAIKVF40**
**C9orf72/**	** 1 5 10**
**Q96LT7**	**241AEKVNKIVRTLCLFLTPAER260**
	** 1 12**
	**241AEKVNKIVRTLCLFLTPAER260**
**IQ-Like Motif**	
**SQSTM1/**	**63FQAHYRDEDGDLVA77**
**Q13501**	

Legend: Green indicates relevant hydrophobic aa. FUS, Fused in sarcoma; C9orf72, Chromosome 9 open reading frame 72; SQSTM1, Sequestosome-1; tdp-43, TAR DNA-binding protein 43 (TDP-43); TBK1, TANK-binding kinase 1. Note: Green, critical hydrophobic amino acids; TGM1, transglutaminase 1; TGM2, transglutaminase 2; TGM3, transglutaminase 3.

**Table 3:** Calmodulin binding domains and motifs in Huntington’s risk proteins.

Protein/Uniprot	CaMBD Sequence/Binding Motif
	** 1 12**
**Huntingtin/**	**177NGAPRSLRAALWRFAELAHLVR197**
**P42858**	** 1 8 14**
	**177NGAPRSLRAALWRFAELAHLVR197**
	** 1 5 10**
	**177NGAPRSLRAALWRFAELAHLVR197**
	** 1 12**
**Huntingtin/**	**2535PLKALDTRFGRKLSIIRGIV2554**
**P42858**	** 1 8 14**
	**2535PLKALDTRFGRKLSIIRGIV2554**
	** 1 12**
	**2535PLKALDTRFGRKLSIIRGIV2554**
	** 1 16**
	**2535PLKALDTRFGRKLSIIRGIV2554**
	** 1 12**
	**2535PLKALDTRFGRKLSIIRGIV2554**
	** 1 16**
**TGM2/**	**414KSINRSLIVGLKISTKSVGR433**
**P21980**	** 1 12**
	**414KSINRSLIVGLKISTKSVGR433**
	** 1 5 10**
	**414KSINRSLIVGLKISTKSVGR433**
	** 1 12**
**TGM2/**	**665VVNFESDKLKAVKGFRNV683**
**P21980**	** 1 8 14**
	**665VVNFESDKLKAVKGFRNV683**
	** 1 10**
	**665VVNFESDKLKAVKGFRNV683**
	** 1 12**
	**665VVNFESDKLKAVKGFRNV683**
	** 1 10**
	**665VVNFESDKLKAVKGFRNV683**
	** 1 12**
**TGM1/**	**607RRTVKLHLYLSVTFYTGVS625**
**P22735**	** 1 5 10**
	**607RRTVKLHLYLSVTFYTGVS625**
	** 1 10**
**TGM3/**	**493LAVGKEVNLVLLLKNLSRDT512**
**Q08188**	** 1 12**
	**493LAVGKEVNLVLLLKNLSRDT512**
	** 1 16**
	**493LAVGKEVNLVLLLKNLSRDT512**
	** 1 5 10**
	**493LAVGKEVNLVLLLKNLSRDT512**
	** 1 14**
	**493LAVGKEVNLVLLLKNLSRDT512**
	** 1 5 10**
	**493LAVGKEVNLVLLLKNLSRDT512**

Legend: Green, critical hydrophobic amino acids; TGM1, transglutaminase 1; TGM2, transglutaminase 2; TGM3, transglutaminase 3.

## Data Availability

Not applicable.

## Abbreviations

AchR	Acetylcholine receptor
AD	Alzheimer’s disease
ALS	amyotrophic lateral sclerosis
APOE	apolipoprotein E
BD	Batten disease
BIN1	Bridging Integrator 1
C9orf72	chromosome 9 open reading frame 72
CaM	calmodulin
CaMBD	calmodulin binding domain
CaMBP	calmodulin binding protein
CD33	myeloid cell surface antigen CD33
CH3L1/YKL-40	chitinase-3-like protein I
CHCHD10	Coiled-coil-helix-coiled-coil-helix domain-containing protein 10
CLU	clusterin
CR1	complement receptor type 1
D2R	D2-dopamine receptor
EPHA1	ephrin type-A receptor 1
FTD	frontotemporal dementia
FUS	fused in sarcoma RNA binding protein
GBA	glucocerebrosidase
GRN	progranulin
HD	Huntington’s disease
LBD	dementia with Lewy bodies
LRRK2	leucine-rich repeat kinase 2
MS	multiple sclerosis
MS4A	membrane-spanning 4-domains subfamily A
NCL	neuronal ceroid lipofuscinosis
NLRP3	NACHT, LRR, and PYD domains containing protein 3
PD	Parkinson’s disease
PARK7	Parkinsonism associated deglycase 7
PILRA	paired immunoglobin-like type 2 receptor alpha
PINK1	PTEN induced kinase 1
PP2B	calcineurin
SNCA	α-synuclein SQSTM1, TGM1/2, transglutaminase 1/2
SQSTM1	TGM1/2, transglutaminase 1/2
TMEM175	transmembrane protein 175
TPD-43/TARDBP	TPD-43/TARDBP
TREM2	triggering receptor expressed on myeloid cells 2
VCP	Valosin-containing protein
